# Prevalence of dental anomalies of number in different subphenotypes of isolated cleft palate

**DOI:** 10.1590/2176-9451.19.1.055-059.oar

**Published:** 2014

**Authors:** João Paulo Schwartz, Daniele Salazar Somensi, Priscila Yoshizaki, Luciana Laís Savero Reis, Rita de Cássia Moura Carvalho Lauris, Omar Gabriel da Silva Filho, Gisele Dalbén, Daniela Gamba Garib

**Affiliations:** 1 Specialist in Orthodontics, Hospital for Rehabilitation of Craniofacial Anomalies - São Paulo University (HRAC-USP).; 2 MSc in Orthodontics, HRAC-USP.; 3 PhD in Pediatric Dentistry, HRAC-USP.; 4 Full professor and assistant professor of Orthodontics, School of Dentistry - University of São Paulo/Bauru.

**Keywords:** Cleft palate, Tooth abnormalities, Panoramic radiograph

## Abstract

**Objective:**

This study aimed at carrying out a radiographic analysis on the prevalence of dental anomalies of number (agenesis and supernumerary teeth) in permanent dentition, in different subphenotypes of isolated cleft palate pre-adolescent patients.

**Methods:**

Panoramic radiographs of 300 patients aged between 9 and 12 years, with cleft palate and enrolled in a single treatment center, were retrospectively analyzed. The sample was divided into two groups according to the extension/severity of the cleft palate: complete and incomplete . The chi-square test was used for intergroup comparison regarding the prevalence of the investigated dental anomalies (P < 0.05).

**Results:**

Agenesis was found in 34.14% of patients with complete cleft palate and in 30.27% of patients with incomplete cleft palate. Supernumerary teeth were found in 2.43% of patients with complete cleft palate and in 0.91% of patients with incomplete cleft palate. No statistically significant difference was found between groups with regard to the prevalence of agenesis and supernumerary teeth. There was no difference in cleft prevalence between genders within each study group.

**Conclusion:**

The prevalence of dental anomalies of number in pre-adolescents with cleft palate was higher than that reported for the general population. The severity of cleft palate did not seem to be associated with the prevalence of dental anomalies of number.

## Abstract (pt)

**Objetivo:**

o propósito deste estudo foi avaliar radiograficamente a prevalência das anomalias dentárias de número (agenesias e supranumerários), na dentição permanente, em diferentes subfenótipos da fissura isolada de palato, em pacientes pré-adolescentes.

**Métodos:**

foram investigadas, de forma retrospectiva, 300 radiografias panorâmicas de pacientes com fissura palatina (pós-forame), de 9 a 12 anos de idade, matriculados em um mesmo centro. A amostra foi dividida em dois grupos de acordo com a extensão/gravidade da fissura palatina: completa e incompleta. O teste qui-quadrado foi utilizado para comparação intergrupos das prevalências de anomalias avaliadas (p < 0,05).

**Resultados:**

a agenesia dentária foi encontrada em 34,14% dos pacientes com fissura pós-forame completa e em 30,27% com fissura pós-forame incompleta. A prevalência de dentes supranumerários correspondeu a 2,43% nos pacientes com fissura palatina completa e a 0,91% no grupo com fissura palatina incompleta. Não houve diferença estatística significativa entre os grupos quanto à prevalência de agenesias dentárias e supranumerários. Não se observou diferença sexual quanto à prevalência de fissura dentro de cada grupo de estudo.

**Conclusão:**

os pacientes pré-adolescentes com fissura palatina apresentam maior prevalência de anomalias dentárias em relação à população em geral. A gravidade da fissura palatina parece não se associar com a prevalência de anomalias dentárias de número.

## INTRODUCTION

The embryonic explanation for isolated cleft palate is the lack of fusion of the palatal shelves that form the secondary palate. In this type of cleft, the palatine processes do not fuse neither in the midline nor with the nasal septum, keeping the communication between oral and nasal cavities, while the formation of lips and alveolar ridge is processed normally.^1^

**Figure 1:**
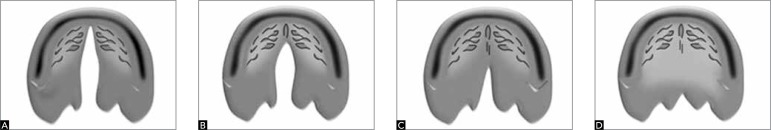
Complete cleft palate invariably extends from the incisive foramen to the uvula (A). Incomplete cleft palate involves the posterior region of the palate without reaching the incisive foramen (B); affects the soft palate and part of the hard palate (C); or may affect only the soft palate (D).

Isolated cleft palate may be complete or incomplete.^1^ It is considered complete when it affects the hard and soft palate, extending to the incisive foramen (Fig 1A). On the other hand, it is considered incomplete when it partially affects the soft and/or hard palate, not reaching the incisive foramen (Figs 1B, 1C and 1D).

The prevalence of cleft lip and palate is approximately 1:1000 births.^1^ In general, individuals of Asian descent have higher prevalence while those of African descent have lower prevalence when compared to Caucasian individuals.^2^ The etiology of cleft lip and palate is complex, with multifactorial causality, in which case both genetic and environmental factors play a major role in determining the malformation.^3^ From an embryological standpoint, cleft palate is a disorder that differs from cleft lip and palate. Differences in epidemiology and etiologic factors have also been reported in the literature.^3^

Similarly to the general population, odontogenic disorders are also found in patients with clefts. It is assumed that cleft and dental anomalies present a common or inter-related genetic origin, considering the high prevalence of dental anomalies in cleft patients .^4^ In other words, patients with clefts present more incidence of dental anomalies than individuals without clefts.^5^ Moreover, the prevalence of dental anomalies seems to be related to the extension/severity of cleft lip and palate.^6^

The diagnosis of dental anomalies of number is essential to define the treatment plan in the rehabilitation process of patients with cleft palate, either orthodontic, with prosthesis or implants. The purpose of this study was to radiographically assess the prevalence of dental anomalies of number in different subphenotypes of isolated cleft palate.

## MATERIAL AND METHODS

This study was conducted in the department of Orthodontics at the Hospital for Rehabilitation of Craniofacial Anomalies, University of São Paulo (HRAC-USP), after approval by the respective Institutional Review Board. The study analyzed the radiographs of 300 patients from the HRAC-USP files. The sample comprised 117 (39%) males and 183 (61%) females who were in late mixed dentition (second transitional period of mixed dentition according to the Van der Linden classification) and early permanent dentition. The patients aged between 9 and 12 years old (chronological age). At this age, the third molars were excluded from the evaluation.

The total sample was divided into two study groups, according to the extension of cleft palate indicated in the medical records of patients: Group 1 - complete cleft palate; and Group 2 - incomplete cleft palate.

The occurrence of permanent teeth agenesis and supernumerary permanent teeth was evaluated in panoramic radiographs by a calibrated observer with the aid of a film viewer in a room with appropriate lightening. The study included only radiographs with good technical quality that allowed good visualization of teeth, erupted or not, and their surrounding structures.

After the prevalence of dental anomalies was calculated in each study group, the chi-square test was used for comparison. It was also used to verify intragroup differences in the prevalence of anomalies between sexes. The results were considered at a significance level of 5%.

## RESULTS

Out of the 300 patients analyzed, 82 (27.33%) had complete cleft palate, whereas 218 (72.66%) had incomplete cleft palate. Among individuals with complete cleft palate, 31 (37.8%) were males and 51 (62.19%) females. As for patients with incomplete cleft palate, 86 (39.44%) were males and 132 (60.55%) females. In both groups, the proportion between male and female was approximately 1:2.

The prevalence of dental anomalies in Group 1 (complete cleft palate) and Group 2 (incomplete cleft palate) is expressed in percentage and presented in Table 1. In Group 1, tooth agenesis of permanent teeth excluding the third molars was observed in 28 (34.14%) patients,while supernumerary teeth were found in 2 (2.43%). In Group 2, tooth agenesis was observed in 66 (30.27%) patients, while supernumerary teeth was found in 2 (0.91%). No significant difference in the prevalence of hypodontia and supernumerary teeth was found between Groups 1 and 2 (Table 1). Additionally, there was no difference between groups in the prevalence of agenesis for the most commonly affected teeth: second premolars and maxillary lateral incisors (Table 1).

**Table 1:** Prevalence of dental anomalies in groups 1 and 2, and intergroup comparison results (chi-square test).

	G1 + G2	G1	G2	χ^2^	p
Hypodontia	31.33%	34.14%	30.27%	0.0064	0.9359
Supernumerary	1.33%	2.43%	0.91%	0.0000	1.0000
Hypodontia MxLI	8.50%	7.92%	8.71%	1.9340	0.1644
Hypodontia Mx2P	7.50%	9.14%	6.88%	0.0000	1.0000
Hypodontia Md2P	8.66%	6.70%	9.40%	0.2545	0.6139

* MxLI = maxillary lateral incisor, Mx2P = maxillary second premolar, Md2P = mandibular second premolar.

**Figure 2:**
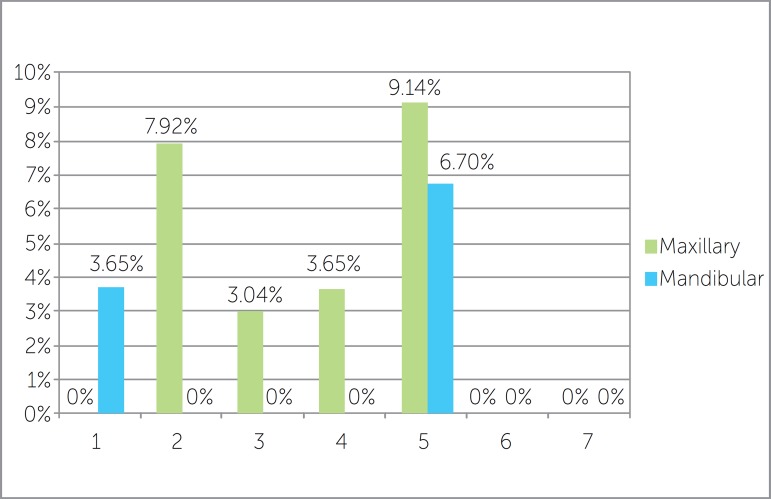
Prevalence of agenesis of each permanent tooth (excluding third molars) in the complete cleft palate group (group 1).

**Figure 3:**
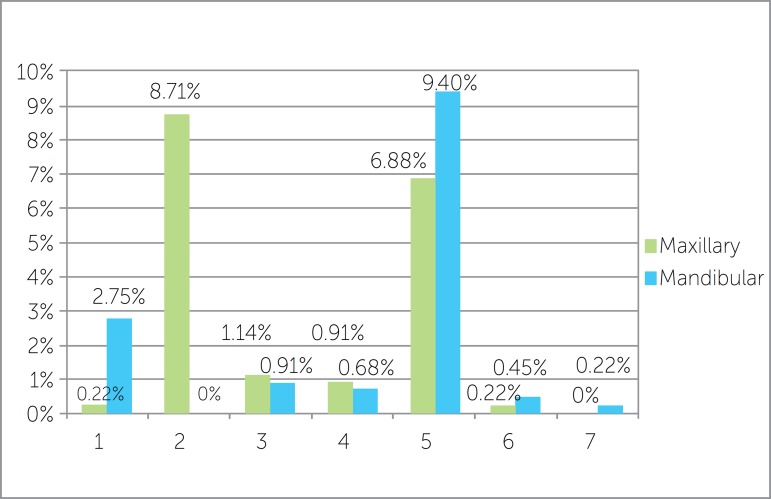
Prevalence of agenesis of each permanent tooth (excluding third molars) in the incomplete cleft palate group (group 2).

Figures 2 and 3 show the prevalence of agenesis of each permanent tooth in Groups 1 and 2, respectively. With regard to supernumerary teeth, in Group 1 they were found in the region of the maxillary left lateral incisor and the maxillary left second premolar, while in Group 2 supernumerary teeth were observed in the region of the maxillary right lateral incisor and between the central incisors (mesiodens).

The distribution of dental anomalies according to sex is shown in Table 2. In both groups, there was no statistically significant difference between males and females for the prevalence of tooth agenesis and supernumerary teeth (Table 2).

**Table 2:** Prevalence of dental anomalies according to sex and intragroup comparison results (chi-square test).

	Complete cleft palate				Incomplete cleft palate			
	Female	Male	χ^2^	p	Female	Male	χ^2^	p
Hypodontia	35.29%	32.25%	0.0390	0.8434	32.57%	26.74%	0.2798	0.5968
Supernumerary	0%	6.45%	1.0960	0.2952	0%	2.32%	1.0300	0.3101

## DISCUSSION

Cleft palate is more common among female patients.^1,7^ In this study, both complete and incomplete cleft palate prevailed in females. In individuals without clefts, excluding the third molars, the prevalence of tooth agenesis in the population varies from 4.3% to 7.8%, primarily affecting the mandibular second premolar, followed by the maxillary lateral incisor and maxillary second premolar.^8^ The present results show that the prevalence of tooth agenesis is much higher in individuals with cleft palate than in the general population.

According to this study, the prevalence of hypodontia of permanent teeth, excluding the third molars, in patients with cleft palate was 31.33% and was similar to that reported in the literature for patients with these malformations. Previous studies reported a prevalence of permanent tooth agenesis in patients with cleft lip and palate of 25.5% to 33% in Czech patients,^9^ 30% in Swedish patients,^10^ 25% to 40%^11^ and 33% in Finnish patients^12^ and 28.5% in Norwegian patients.^13^ Another previous study reported that tooth agenesis was observed more frequently in patients with complete cleft palate than in patients with incomplete cleft palate.^11^ However, our study did not find any difference in the occurrence of hypodontia according to the subphenotypes (Table 1).

As for the subphenotypes, in both complete and incomplete cleft palate the teeth most affected by hypodontia were the maxillary and mandibular second premolars as well as maxillary lateral incisors, particularly the maxillary second premolars in complete cleft palate and the mandibular second premolars in incomplete cleft palate (Figs 2 and 3). These data are in accordance with other reports in the literature,^9,10,14^ and are similar to those found for the general population.^4^

The prevalence of supernumerary teeth in patients with cleft palate found in our study is similar to the prevalence found for the general population, with no significant difference between complete and incomplete cleft subphenotypes. Supernumerary teeth were prevalent in 2.43% of group 1 and 0.91% of group 2 (Table 1). In patients with complete palatine cleft, supernumerary teeth were found in the region of maxillary left lateral incisors and maxillary left second premolars. In patients with incomplete cleft palate, they were found in the region of maxillary right lateral incisors and between the central incisors (mesiodens). The prevalence of supernumerary teeth in adolescents without clefts is 1% to 2%.^15^ A previous investigation found no supernumerary teeth in patients with isolated cleft palate.^10^

A recent study found that the presence of dental anomalies may represent an additional clinical marker for oral clefts, suggesting a common genetic origin for these anomalies.^16^ The development of tooth germs and the occurrence of cleft palate are closely related during embryological development, both anatomically and chronologically, and many studies have reported the manifestation of dental anomalies associated with various forms of cleft lip, cleft palate or both.^16^ It has been proposed that individuals with cleft have higher prevalence of dental anomalies than the general population, and that the severity of the malformations seems to be directly related to the extension of the cleft.^16^ In this study, the prevalence of tooth agenesis and the total prevalence of dental anomalies, except for supernumerary teeth, was slightly higher in female patients, although no statistically significant difference was found. The same was observed for the occurrence of complete and incomplete isolated cleft palate.

## CONCLUSION

The prevalence of dental anomalies of number seems not to be related to the subphenotypes of cleft palate. Individuals with complete and incomplete cleft palate showed a similar prevalence of permanent tooth agenesis and supernumerary teeth. Further studies are necessary to determine the exact inter-relation between cleft palate and the prevalence of other dental anomalies.
